# Biomechanical Evaluation of Stress Distribution in Equicrestal and Sub-crestally Placed, Platform-Switched Morse Taper Dental Implants in D3 Bone: Finite Element Analysis

**DOI:** 10.7759/cureus.24591

**Published:** 2022-04-29

**Authors:** Yashaswini Ellendula, Anam Chandra Sekar, Sandeep Nalla, Ram B Basany, Kunchala Sailasri, Ashwini Thandu

**Affiliations:** 1 Department of Prosthodontics and Crown & Bridge, SVS Institute of Dental Sciences, Mahabubnagar, IND; 2 Department of Prosthodontics and Crown & Bridge, SVS Institute Of Dental Sciences, Mahabubnagar, IND

**Keywords:** fea, platform-switched implant, morse taper implant, sub-crestal, equi-crestal

## Abstract

Aim

The aim of the study was to assess the effect of implant placement depth on stress distribution in bone around a platform-switched and Morse taper dental implants placed at the equi-crestal and 1 mm and 2 mm sub-crestal levels in a D3 bone using the 3D finite element analysis.

Methodology

A mechanical model of a partially edentulous maxilla was generated from a computerized tomography (CT) scan of an edentulous patient, as it can give exact bony contours of cortical bone. Also, from accurate geometric measurements obtained from the manufacturer, 3D models of Morse taper and platform-switched implants were manually drawn. The implant and bone models were then superimposed to simulate implant insertion in bone. Three implant positioning levels such as the equi-crestal, 1 mm sub-crestal, and 2 mm sub-crestal models were created, and meshing was done to create the number of elements for distribution of applying loads. The elastic properties of cortical bone and implant, such as Young's modulus and Poisson's ratio (µ), were determined. A load (axial and oblique) of 200N that simulated masticatory force was applied.

Results

On comparing stresses within the bone around the equi-crestal and 1 mm and 2 mm sub-crestal implants, it was observed that the maximum stresses were seen within cortical bone around the equi-crestally placed implant (21.694), the least in the 2 mm sub-crestally placed implant (18.85), and intermediate stresses were seen within the 1 mm sub-crestally placed implant (18.876).

Conclusion

Sub-crestal (1-2mm) placement of a Morse taper and a platform-switched implant is recommended for long-term success, as maximum von Mises stresses were found within cortical bone around the equi-crestal implant followed by the 1 mm sub-crestal implant and then the 2 mm sub-crestal implant.

## Introduction

Dental treatment today focuses on restoring the patient's health, comfort, and appearance to his or her pre-existing condition, with implant dentistry. Regardless of stomatognathic system degeneration, disease, or injury, this goal can be accomplished.

Sufficient bone volume and density are required for successful oral implants. Practitioners frequently encounter anatomic variations in the premolar and molar areas when using osseointegrated dental implants for edentulous patients, one of which is the maxillary sinus. Surgical placement of an implant in the maxilla will be difficult due to an insufficient posterior alveolus, increased pneumatization of the maxillary sinus, and the sinus's close proximity to the crestal bone [1].

In literature reviews of clinical studies from 1981 to 2001, it has been reported that the poorest bone density can lead to a 16% decrease in implant survival and has been reported as low as 40% [2]. Bone density directly influences the percentage of implant bone surface contact (BIC), which accounts for the force transmission and distribution to the bone.

Bone quality is often determined by the arch position. The densest bone is usually observed in the anterior mandible, and the least dense bone is typically found in the posterior maxilla. In the posterior maxilla, where forces are greater and bone density is lower, clinical failure rates are highest.

In comparison to D1 & D2 bone densities, D3 bone has the least bone-implant contact. In case of poor bone density, stress patterns migrate further toward the implant apex. The interaction between the implant and the bone differs with different geometry of implants [3].

One of the most important variables determining the implant's primary stability and ability to tolerate loads during or after osseointegration is its design. A dental implant’s primary stability is determined by the implant design, surgical technique, and bone properties at the implant site. Hence, the development of fibrous tissue at the bone-implant interface during healing and loading may be minimized with high primary stability, resulting in reduced micromotions and adverse tissue reactions [4].

The advantages of Morse taper dental implant joints associated with platform switching include preserving the soft tissue profile, lowering the incidence of bone loss, and, ultimately, reducing the onset and rate of marginal periimplantitis associated with the implant-abutment platform. These implants should be placed 1 to 2 mm below the bone crest, according to the manufacturer's instructions, to optimize the maintenance of the peri-implant soft tissues surrounding the cervical third of the implant [5].

Several methods, such as strain gauges, photoelastic models, and finite element analysis (FEA), have been used to investigate the relationship between peri-implant bone remodeling, implant design, and loading. Considering the experimental limitations of in vivo studies, FEA has played an important role in the study of the biomechanics of the implant and surrounding bone, as well as in predicting the success of implant systems in various clinical situations [6].

As there are few studies on the comparison of stresses within the cortical and cancellous bone around the equi-crestal and different levels of sub-crestal implant positioning, the present study was conducted to evaluate the long-term success of the implant by studying the distribution and magnitude of stresses through the D3 bone tissue surrounding platform-switched and Morse taper dental implants at different positioning levels such as equi-crestal, 1 mm sub-crestal, and 2 mm sub-crestal.

## Materials and methods

A CAD model of the CT scan reproducing a partially edentulous maxilla having a cancellous core of D3 bone surrounded by a 2.5 mm thick cortical layer and CAD models of the K3Pro® dental implants (Argon Medical Productions & Vertriebs, Gesellschaft mbh & Co. KG, Bingen am Rhein, Germany) were created.

The software programs used are 3D Slicer (Harvard University, Cambridge, MA) for the creation of the CAD model from the spiral CT data, CATIA version 5.0 (Dassault Systèmes SE, Vélizy-Villacoublay, France) for 3D modeling, Hypermesh version 17.0 (Altair, Troy, Michigan) for meshing, and OptiStruct version 17.0 (Altair) for solving.

**Figure 1:**
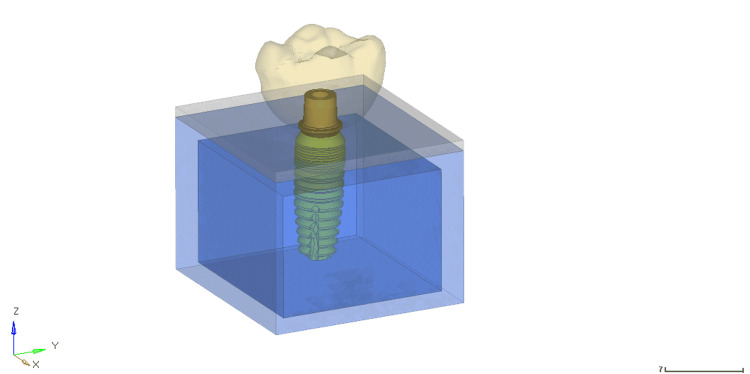
Superimposed geometric model of implant, abutment, bone, mucosa, and crown

Dimensions and properties for cortical bone, trabecular bone, and titanium implants were assigned to models (Figure 1). Each solid component with isotropic, homogeneous, and linearly elastic behavior was assigned its Young's modulus and Poisson's ratio (Table 1) [7].

**Table 1:** Material properties assigned to models

Material	Young’s modulus (E)	Poison’s ratio (µ)
Cortical bone	14.8	0.3
Cancellous bone	0.69	0.3
Titanium	110	0.35
Porcelain crown	82.8	0.35
Mucosa	10	0.40

**Figure 2:**
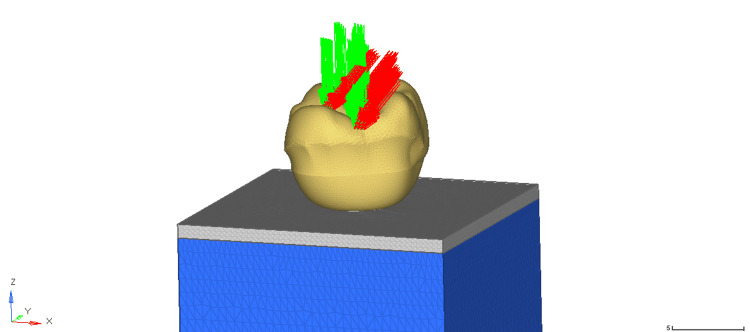
Application of axial and oblique load (200N) on the crown

A load (axial and oblique) of 200N was applied on the occlusal surface of the first molar (Figure 2), and von Mises stresses around the implant and within the compact and cancellous bone were interpreted [8].

**Figure 3:**
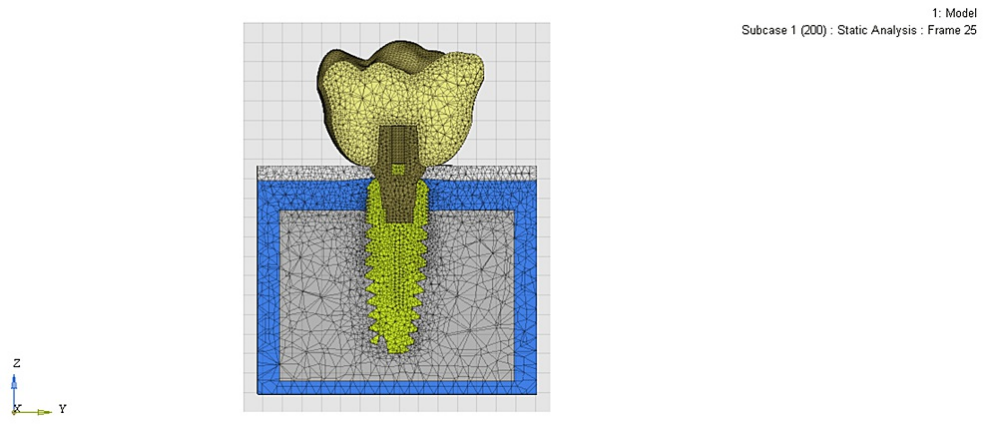
Mesh model of the equi-crestally placed implant

**Figure 4:**
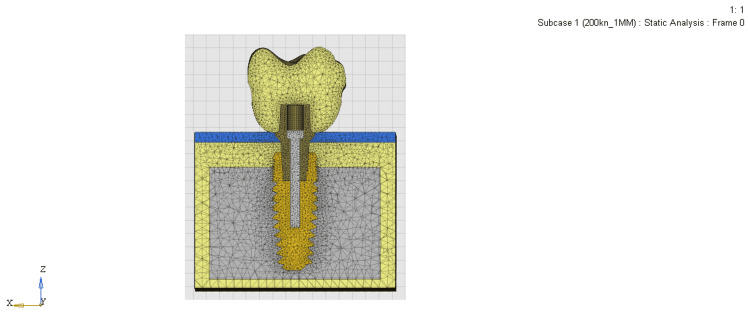
Mesh model of the 1 mm sub-crestally placed implant

**Figure 5:**
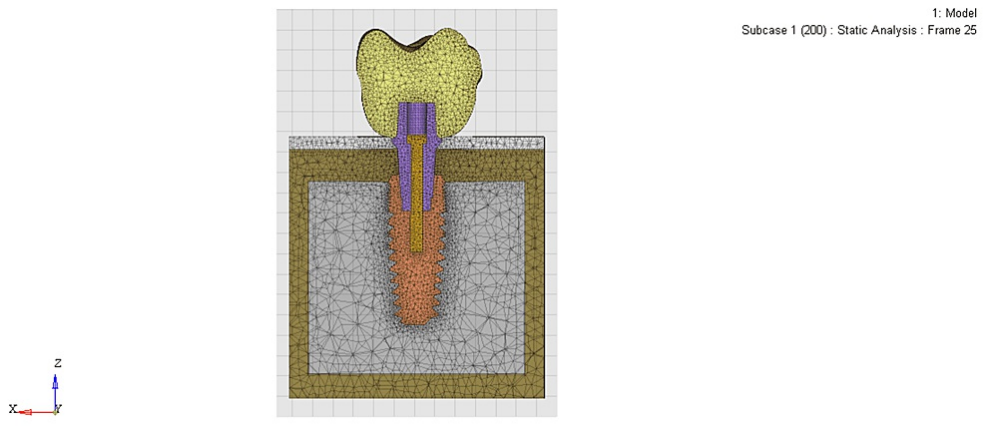
Mesh model of the 2 mm sub-crestally placed implant

After the final models were obtained (Table 2), OptiStruct Version 17.0 software was used for simulating and solving the loading condition and post-processing analysis of the models (Figures 3-5). The results of the mathematical solutions were converted into visual results and expressed in color gradients ranging from red to blue. Blue is for minimum and red is for maximum stress. Shades in between are the variation of stress or displacement from minimum to maximum. The stress values at various points in the three models were collected and compared, and points of greatest stress were identified by the von Mises equivalent stress levels.

**Table 2:** Three sets of models

Sl. No	Models
1.	K3Pro implant in the maxillary molar region positioned equi-crestally with vertical and oblique load applied.
2.	K3Pro implant in the maxillary molar region positioned 1 mm sub-crestally with vertical and oblique load applied.
3.	K3Pro implant in the maxillary molar region positioned 2 mm sub-crestally with vertical and oblique load applied.

## Results

**Figure 6:**
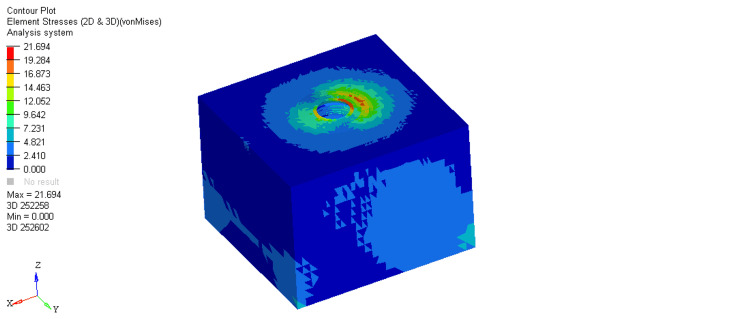
Stress patterns generated within the cortical and cancellous bone around an implant positioned equi-crestally under a load of 200N

**Figure 7:**
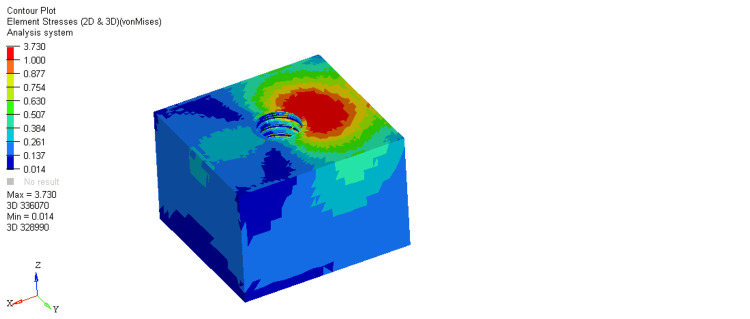
Stress patterns generated within the cortical and cancellous bone around an implant positioned equi-crestally under a load of 200N

**Figure 8:**
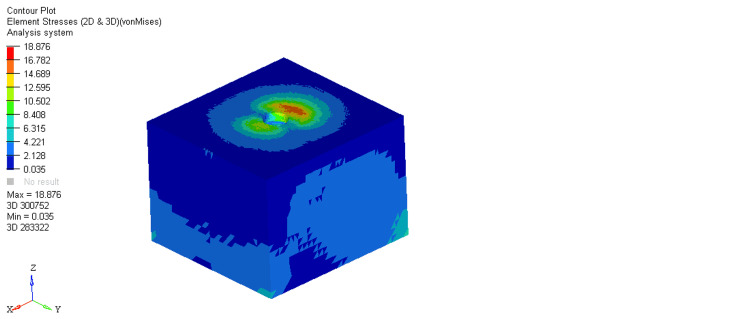
Stress patterns generated within the cortical and cancellous bone around an implant positioned 1 mm sub-crestally under a load of 200N

**Figure 9:**
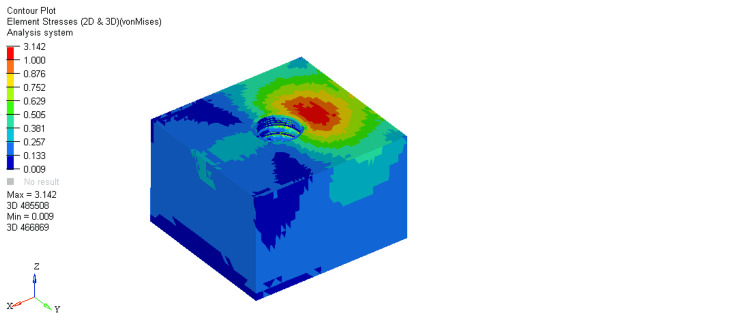
Stress patterns generated within the cortical and cancellous bone around an implant positioned 1 mm sub-crestally under a load of 200N

**Figure 10:**
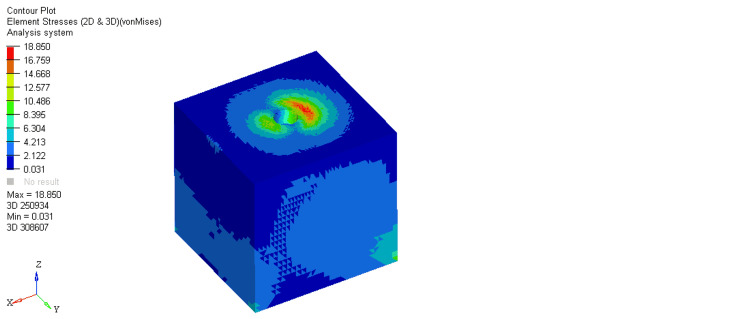
Stress patterns generated within the cortical and cancellous bone around an implant positioned 2 mm sub-crestally under a load of 200N

**Figure 11:**
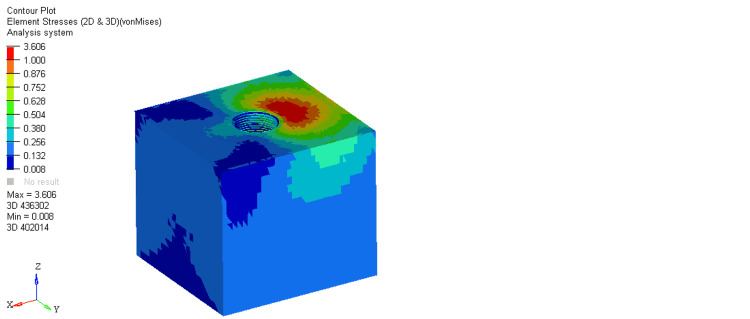
Stress patterns generated within the cortical and cancellous bone around an implant positioned 2 mm sub-crestally under a load of 200N

On comparing stresses within the bone around the equi-crestal and 1 mm and 2 mm sub-crestal implants, it was observed that the maximum stresses were seen within cortical bone around the equi-crestally placed implant, the least in the 2 mm sub-crestally placed implant, and intermediate stresses were seen within the 1 mm sub-crestally placed implant (Figures 6-11). On comparing cortical and cancellous bone, the least stresses were seen within the cancellous bone in all three models (equi-crestal, 1 mm sub-crestal, and 2 mm sub-crestal) of implants (Tables 3-6).

**Table 3:** Stress patterns generated within the cortical and cancellous bone around an implant positioned equi-crestally under a load of 200N

Bone type	Load	Maximum stress	Minimum stress
Cortical	200N	21.694	2.41
cancellous	200N	3.730	0.014

**Table 4:** Stress patterns generated within the cortical and cancellous bone around an implant positioned 1 mm sub-crestally under a load of 200N

Bone type	Load	Maximum stress	Minimum stress
Cortical	200N	18.876	0.035
Cancellous	200N	3.142	0.009

**Table 5:** Stress patterns generated within the cortical and cancellous bone around an implant positioned 2 mm sub-crestally under a load of 200N

Bone type	Load	Maximum stress	Minimum stress
Cortical	200N	18.85	0.031
Cancellous	200N	3.606	0.008

**Table 6:** Comparison of stresses within the bone around the equi-crestal and 1 mm and 2 mm sub-crestal implants

Bone type	Equi-crestal		1mm sub-crestal		2mm sub-crestal	
	Max	Min	Max	Min	Max	Min
Cortical	21.694	2.41	18.876	0.035	18.85	0.031
Cancellous	3.730	0.014	3.142	0.009	3.606	0.008

## Discussion

Improvement and innovations in dental implant design strongly contributed to the development of clinical implant dentistry, resulting in new concepts and even milestone-like changes in the clinical protocol [9].

Implant design and load distribution at the implant-bone interface are important factors to take into consideration. Load transfer at the implant-bone interface is influenced by a variety of factors, including the type of loading, properties of the material of the implant and prosthesis, implant geometry, surface morphology, implant design (diameter and length), quantity of surrounding bone, and nature of the bone-implant interface. As per research, the problem of posterior implant fracture appears to be the result of an elevated risk of overload [10].

There is a higher rate of implant failure in the maxilla due to the amount and quality of bone. In D3 bone, a thin layer of cortical bone surrounds a core of dense trabecular bone [11]. Since softening and remineralization are normal events in bone healing, implant stability will change over time.

D3 bone usually requires one implant per tooth in the posterior region, where fewer implants can be needed in the denser bone area.

Jokstad et al. mentioned the development of internal connections that yielded improved esthetic results and mechanical stability regarding dental implant joints [12].

Two popular implant-abutment connection designs are the internal hexagonal and the Morse taper. Morse taper implant-abutment connections are distinguished by the internal joint design that connects two conical structures. This connection was developed by Stephen A. Morse in 1864, and it was used to connect drilling machines to a detachable rotating drill bit. Tightening a conical "male" abutment onto a conical "female" implant is what it is all about. The internally tapered design creates significant friction [13] due to the high propensity for parallelism between the two components within the joint space. A true Morse taper can be found at 2° and 4° and has an exceptional self-locking characteristic without the need for threads.

Internally stable design allows combining narrower abutment platforms with platform switching. Clinically, platform-switching abutments have been shown to reduce marginal bone loss and provide additional space for soft tissue development and maintenance [13].

One of the criteria used to assess the success of dental implants is the peri-implant bone level. For preserving the integrity of gingival margins and interdental papillae, it is a prerequisite [14].

A Morse taper implant system with platform switching allows for a better connection between both the implant and the intervening abutment, resulting in longer healing and health in the surrounding tissues.

Stress and strain have been shown to be important parameters for crestal bone maintenance and implant survival. The study of these stresses cannot be done on the patient directly. So the finite element analysis (FEA) method can be used to evaluate these stresses on the implant. If these stresses are more favorable under desired values, they can be used in the patient [15].

The finite element method (FEM), which originated from aerospace engineering, has been used for five decades for numerical stress analysis. FEM has become incredibly popular for stress analysis owing to its excellent simplicity and reproducibility. The use of FEM as an analytical method eliminates the necessity of performing the difficult task of making direct experimental measurements. FEM offers a more detailed evaluation of the complete state of the stresses [15].

Model considerations

From a computerized tomography (CT) scan, a mechanical model of an edentulous maxilla was generated. The CT scan can provide exact bony contours of cortical bone. Also, from precise geometric measurements obtained from the manufacturers, 3D models of morse taper and platform switching implants were manually drawn. The implant and bone models are then superimposed to simulate implant placement in bone. Three implant positioning levels, the equi-crestal, 1 mm sub-crestal, and 2 mm sub-crestal models, were created and meshing was done to create the number of elements for distribution of applying loads. The size of the bone segment was chosen so that the end effects (stress extended to the ends of the bone segment) should not impinge on the results at the region of interest. At the distal end of the bone, boundary conditions were applied to simulate muscle forces.

Material properties

Cortical bone, cancellous bone, and the implant with abutment were all assumed to be linearly elastic, homogeneous, and isotropic [11]. The corresponding elastic properties, such as Young’s modulus and Poisson’s ratio of cortical bone and implant, were determined according to a literature survey [7].

Implant

The K3Pro® implant system is a commercially pure titanium implant with a unique conical press-fit connection that connects the implant and the abutment with a 1.5° (total 3°) Morse taper and platform-switching. This gives an optimal load displacement from the abutment to the implant. The Morse taper of 1.5° connection also offers a bacterial seal that is free from micro-movement and micro-gaps. Consequently, the implant maintains bone height, tissue health, and long-term stability.

The proven philosophy of K3Pro® is the placement of the implant sub-crestally. By virtue of the fully bacteria-proof and micro-motion-free cone as well as the sloping shoulder, the implant heals deeply into the bone. The dimensions of the implant used in the present study were: length - 9 mm and width - 5 mm.

Todescan et al. found that crestal bone resorption around more deeply placed external hex implants was higher and increased with time [16], whereas Pontes et al. found that the level of internal hex implant placement did not jeopardize the height of the peri-implant ridge [5]. Novaes et al., in a clinical and radiographic study in dogs, where platform-switched implants had been positioned at the level of the crest and 1.5 mm below the crest, found that sub-crestal implants showed better results compared to crestal implants. The sub-crestal position of the implants resulted in bone formation above the implant shoulder [17].

Loading conditions

The magnitude and direction of the loading forces were derived from the study of Rismanchian et al., where 200N of vertical and angled load, simulating the load from the muscles of mastication, was applied [8].

Stress distribution

The result of the present study showed equivalent von Mises stress in three types of implant models during the application of loads. Peri-implant cortical bone stress increased by placing the implant at the level of the bone crest. This is in accordance with Huang et al., who also showed that this increase is more prominent in the supracrestal positions. In contrast, Chu et al. stated that cortical bone stress values decreased by increasing the placement depth. They did not simulate any superstructure, and the force was applied to the abutment [18].

A mechanical distribution of stress occurs primarily in areas where bone contacts the implant. In cortical bone, the percentage of bone contact is significantly higher than in cancellous bone. Maximum stresses are placed on the bone around the implant neck. It was consistently found in the present study. On loading, the stresses generated in the cortical bone surrounding the equi-crestal implant model was 21.694 MPa, which was comparatively more than those generated around the 1 mm and 2 mm sub-crestal implant models (18.876 MPa and 18.85 MPa, respectively).

On comparing the stresses around implants in three different models, it is evident that the stresses are maximum (21.694 MPa) in Model 1 (equi-crestal implant) and minimum (18.85 Mpa) in Model 3 (2 mm sub-crestal implant).

When the evaluation of the stresses was done with respect to the cortical bone in three different models (Figure 6, Figure 8, Figure 10), it is clearly evident that the maximum stresses in the cortical bone (21.694MPa) are again attributed to Model 1 (equi-crestal implant) and the minimum stresses (18.85 MPa) in Model 3 (2 mm sub-crestal implant).

On comparing the stresses with respect to the cancellous bone in three different models (Figure 7, Figure 9, Figure 11), it is evident that the maximum stresses in the cancellous bone (3.730 MPa) in Model 1 (equi-crestal implant) and the minimum stresses (3.142 Mpa) in Model 2 (1 mm sub-crestal implant).

On overall comparison, stresses were seen more in the equi-crestal implant than in 1 mm and 2 mm sub-crestal implant models during the application of loads.

Limitations of the present FEA study

Anisotropic tissues, such as the cortical bone, cancellous bone, and implant, are considered isotropic initially. The load applied was static, rather than the dynamic load encountered during function.

FEA relies on mathematical calculations that simulate the structure within its environment. But living tissues, on the other hand, cannot be contained within set parameters and values since biology does not exist in the same way as set parameters.

## Conclusions

This study evaluated in detail the effect of implant placement depth on stress distribution in bone around platform-switched and Morse taper dental implants placed at the equi-crestal and 1 mm and 2 mm sub-crestal levels in a D3 bone using the 3D FEA.

Within the limitations of this FEA study, we concluded that maximum von Mises stresses were found within cortical bone around the equi-crestal implant followed by the 1 mm sub-crestal implant and then the 2 mm sub-crestal implant. On comparing the cortical and cancellous bone, maximum stresses were found within the cortical bone than in the cancellous bone. Sub-crestal (1-2 mm) placement of a Morse taper and platform-switched implant can be recommended for long-term success.
